# Integrating DHIS2 and R for Enhanced Cholera Surveillance in Lebanon: A Case Study on Improving Data Quality

**DOI:** 10.3390/ijerph22111684

**Published:** 2025-11-06

**Authors:** Abass Toufic Jouny, Hawraa Sweidan, Maryo Baakliny, Nada Ghosn

**Affiliations:** 1 Epidemiological Surveillance Program, Ministry of Public Health, Ras En Nabaa, Beirut 1107, Lebanon; hawraa.sweidan@gmail.com (H.S.);; 2 Mediterranean and Black Sea Programme in Intervention Epidemiology Training (MediPIET), European Centre for Disease Prevention and Control (ECDC), 171 83 Stockholm, Sweden

**Keywords:** cholera outbreak, surveillance system, DHIS2, Lebanon

## Abstract

During the 2022–2023 cholera outbreak in Lebanon, cases were reported through the District Health Information System 2 (DHIS2). We developed automated procedures in R computing language to improve completeness of routinely notified variables, apply case definition criteria, improve geographic accuracy and documentation of laboratory results. We developed R scripts for data cleaning, standardization, and reclassification, plotted epidemic curves and produced maps to display cholera incidence rates and rapid diagnostic test (RDT) coverage by district. We shared the R scripts on GitHub platform for open adaptation and use. Prior to cleaning, missingness reached 99.7% for inpatient status and 17–35% for other key variables. After cleaning, all fields were complete. Initially, 92.8% of cases were notified through DHIS2 as suspected and 7.2% as confirmed. Following reclassification, 40% were classified as suspected, 5.8% as confirmed, and 48.6% with unspecified classification. Laboratory data revealed that 5.8% of cases were culture positive, 2.2% RDT positive, and 65.1% had no documented testing. Among facility-entered cases (n = 5953), 11.4% were reported from a different governorate than the patient’s residence. At the time of the outbreak, the daily maps were generated based on place of residence. Integrating R-based analytics with DHIS2 enhanced data completeness, improved case classification, and enabled more better spatial and laboratory analysis. This combined approach provided a clearer epidemiological picture of the cholera outbreak, supporting data-driven public health decision-making and highlighting the value of integrating analytical tools with routine surveillance systems.

## 1. Introduction

Cholera is an acute diarrheal disease caused by ingestion of food or water contaminated with *Vibrio cholerae* of serogroups O1 or O139. It can lead to severe dehydration and death if untreated, but with prompt case detection, appropriate medical care, and control measures, outbreaks can be rapidly contained [1,2]. Timely, accurate surveillance is therefore critical to guide public health interventions, prevent further spread, and reduce morbidity and mortality [3].

In Lebanon, a cholera outbreak emerged in October 2022 after nearly three decades of absence, highlighting the need for rapid detection and coordinated response [4]. The Ministry of Public Health (MoPH) enhanced the electronic national surveillance system using the District Health Information System 2 (DHIS2), an open-source platform for real-time case notification and outbreak monitoring [5,6]. Through DHIS2, healthcare facilities and laboratories across the country reported suspected and confirmed cases, enabling daily updates of epidemic curve, geographical distribution, and patients characteristics.

Despite this structured surveillance system, the outbreak investigation faced challenges. Healthcare personnel frequently did not strictly follow the standard cholera case definition criteria. In addition, DHIS2 mapped cases according to the reporting health facility rather than the patient’s actual place of residence. Laboratory results, especially from bacterial culture, were not systematically conducted, affecting the accuracy of case classification [7].

To address these gaps, we developed R-based analytical tools to enhance DHIS2 reporting [8,9]. This allowed retrospective application of standardized case definitions, improvement of data completeness and quality, generation of epidemic curves, and automatic mapping of the outbreak both by reporting facility and place of residence. In this manuscript, we describe how the integration of DHIS2 and R enhanced our understanding of the 2022–2023 cholera outbreak in order to improve data handling and analysis for future outbreaks.

Globally, digital platforms such as DHIS2 have been increasingly used to enhance disease surveillance, case-based reporting, and outbreak monitoring [10]. In Nigeria, for example, recent analyses demonstrated the importance of integrating digital health tools and analytics to strengthen routine surveillance across epidemic-prone diseases [11]. Yet, few published reports describe retrospective data-cleaning and analytical re-classification within such systems during a cholera outbreak.

## 2. Materials and Methods

### 2.1. Cholera Case Definition

The national cholera case definition in Lebanon follows the World Health Organization (WHO) guidelines. In countries with no known cholera outbreak, a suspected case is defined as any patient presenting with acute watery diarrhea with severe dehydration or death from acute watery diarrhea. During an outbreak, a suspected case is any person presenting with or dying from acute watery diarrhea. A confirmed case is any suspected case in which *Vibrio cholerae* O1 or O139 is identified through culture or polymerase chain reaction (PCR). These definitions were used to retrospectively reclassify the notified cases during the 2022–2023 cholera outbreak in Lebanon.

### 2.2. Data Preprocessing and Missing Data Imputation

During the 2022–2023 cholera outbreak in Lebanon, surveillance data were managed using the District Health Information System 2 (DHIS2). The system involved a two-stage data entry process: case notification completed by frontline health facilities, and case investigation form completed by the peripheral teams of the Epidemiological Surveillance Program (ESU). The case notification included demographic, clinical information, underlying conditions, vaccination, case management, outcome and laboratory results from health facility laboratories. The case investigation included epidemiological variables, information on underlying conditions, outcome, exposure factors and laboratory test results from reference laboratories. However, the notification stage was not always fully completed, which contributed to a high proportion of missing data in the raw dataset.

To address this, we developed R scripts for automated cleaning and processing of DHIS2 data. This process systematically assessed all variables for completeness and internal consistency, a function not inherently supported within DHIS2, which primarily serves as a data entry and reporting platform. Missing data were completed through triangulation with related fields and information available from the investigation forms (stage 2), such as outcomes, inpatient status, and risk factors. These procedures significantly improved data quality and enabled a complete dataset for further analysis.

### 2.3. Case Classification Based on Clinical and Laboratory Criteria

Initial case classification in DHIS2 relied on the variable called “event”, which allowed health facilities to designate cases as “suspected” or “confirmed” without checking the national cholera case definition. As part of the data cleaning R script, we retrospectively reclassified all the notified cases. We excluded considered symptom frequency, severity (including dehydration, hospitalization, or death), and laboratory results. For reclassification we considered clinical and laboratory categories. Cases with bloody diarrhea were excluded, as they do not meet the WHO suspected cholera case definition.

We grouped cases into four clinical categories: acute watery diarrhea (AWD) with ≥3 episodes per 24 h without complications (dehydration, hospitalisation, death); AWD with complications; AWD with <3 episodes; and cases with insufficient data to classify. These categories allowed evaluation of whether cases met clinical criteria for suspected cholera. The script included plotting a revised epidemic curve to reflect these four categories and highlight the difference between initial notification and reclassification based on case definitions.

We considered positive culture results as the standard confirmatory diagnosis. The rapid diagnostic test (RDT) was not considered confirmatory. Cases that tested positive by both RDT and culture were classified as culture-confirmed. We classified cases into five lab-based categories: culture positive, culture negative, RDT positive, RDT negative, and not tested. We plotted an epidemic curve based on these categories to illustrate the diagnostic certainty of notified cases.

### 2.4. Spatial Distribution and Concordance Between Reporting and Residence

To better understand how cholera cases were distributed across governorates and districts, we compared the location where cases were reported (health facility) with the patient’s recorded place of residence. While DHIS2 collected both types of information, it allowed only map visualization of cases by health facility location.

To improve this, we excluded cases reported by ESU teams (detected during field investigation) and focused only on those entered directly to DHIS2 by healthcare facilities. We then used R to generate additional maps based on patients’ residence.

Each case was categorized as being reported from the same, an adjacent, or a non-adjacent administrative unit compared to the place of residence, at both the governorate and district levels. These differences were summarized and visualized on a map to highlight areas where reporting location and residence differed.

### 2.5. Incidence Rate Mapping and Rapid Diagnostic Test (RDT) Distribution

To assess district-level testing activity and disease burden, we plotted cholera incidence rates and the number of rapid diagnostic tests (RDT) on maps. We calculated incidence rates for each district by dividing the number of reported cholera cases by the estimated district population.

For each district, we extracted the number of performed RDTs from the cleaned surveillance dataset. We used district shapefiles to generate a choropleth map, where incidence rates were displayed using a gradient scale and RDT activity was visualized by overlaying bubbles. This map permitted assessing both burden of disease and diagnostic coverage across the country, highlighting areas with potential gaps in testing.

### 2.6. Data and Code Availability

The R script developed for this study is publicly available on GitHub (https://github.com/jounyabass/dhis2-r-cholera-analysis.git, Accessed on 1 January 2020). Mockup datasets are provided to allow users to test and adapt the code, while actual outputs can only be generated with real surveillance data.

## 3. Results

### 3.1. Data Preprocessing and Missing Data Imputation

A total of 8095 cholera cases were notified in DHIS2 during the 2022–2023 outbreak. We found a high proportion of missing values in several key variables. In stage 1, inpatient status was missing in 99.7% of the records, underlying conditions in 34.7%, illness-related information in 17%, and death outcomes in 34%. Following data cleaning using the data from the investigation form (stage 2), the main variables were rendered complete, with no remaining missing values (Table 1).

### 3.2. Case Classification Based on Clinical and Laboratory Criteria

**Figure 1:**
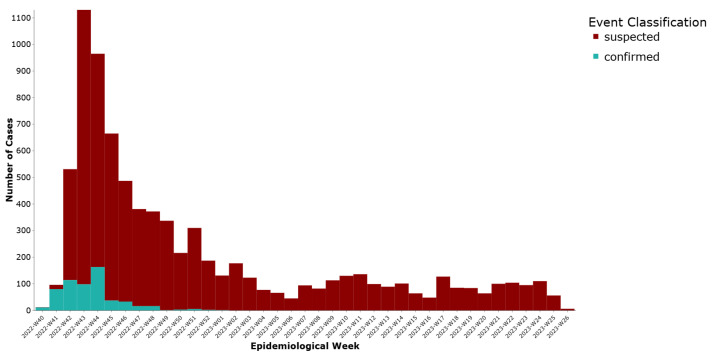
Weekly distribution of cholera cases by initial reporting status, Lebanon, 2022–2023.

A total of 7515 cases (92.8%) were initially reported in DHIS2 as suspected and 580 (7.2%) as confirmed. We noted a peak in weeks 43–44 of 2022 and then a steady decline of notifications (Figure 1).

**Figure 2:**
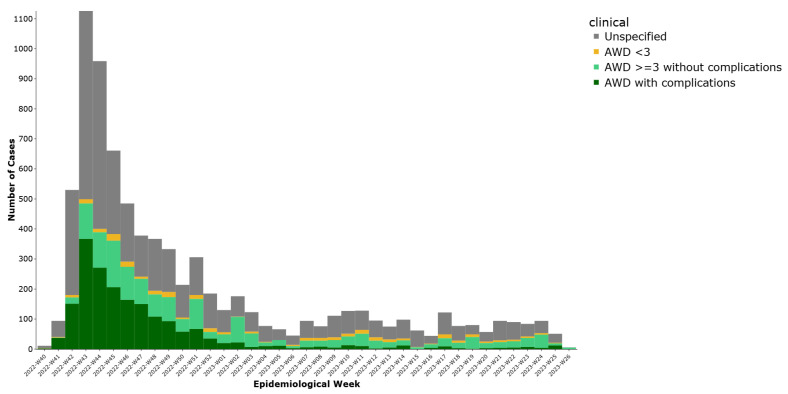
Weekly distribution of cholera cases by clinical diagnosis, Lebanon, 2022–2023.

Based on clinical reclassification (excluding 164 asymptomatic cases), 1565 cases (19.3%) were classified as acute watery diarrhea (AWD) with three or more episodes in 24 h and no complications, while 1895 cases (23.4%) had AWD with complications such as dehydration, hospitalization, or death. Additionally, 296 cases (3.7%) had AWD with fewer than three episodes, and 4175 cases (51.6%) had insufficient data to classify (Figure 2).

**Figure 3:**
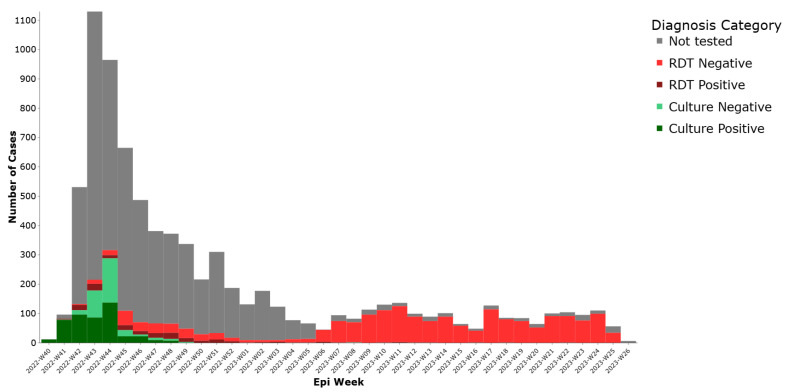
Weekly distribution of cholera cases by lab test results, Lebanon, 2022–2023.

Regarding laboratory classification, (35%) had documented testing. Among these tested, 468 (5.8%) were culture positive, 178 (2.2%) were RDT positive, 304 (3.8%) were culture negative, and 1877 (23.2%) were RDT negative These results are presented in (Figure 3), illustrating the distribution of cases by laboratory confirmation status.

When combining clinical classification with laboratory confirmation, 468 cases (5.8%) were confirmed by culture, 3239 cases (40.0%) met the clinical definition of suspected cholera, 453 cases (5.6%) did not match the case definition, and 3935 cases (48.6%) were unclassifiable due to missing in detailed clinical data (Table 2).

Table 3 compares the initial DHIS2 classification with the reclassified results, showing a substantial improvement in the accuracy of case categorization after applying standardized definitions.

### 3.3. Spatial Distribution and Concordance Between Reporting and Residence

Out of 8095 notified cholera cases, 5953 (73.6%) were entered at the health facility level, while 2142 (26.4%) were entered centrally by ESU teams (detected via investigation, call center, or media/internet scanning). Mapping was restricted to the 5953 cases reported from facilities to allow reliable geographic comparisons between the place of case reporting and patient residence.

**Figure 4:**
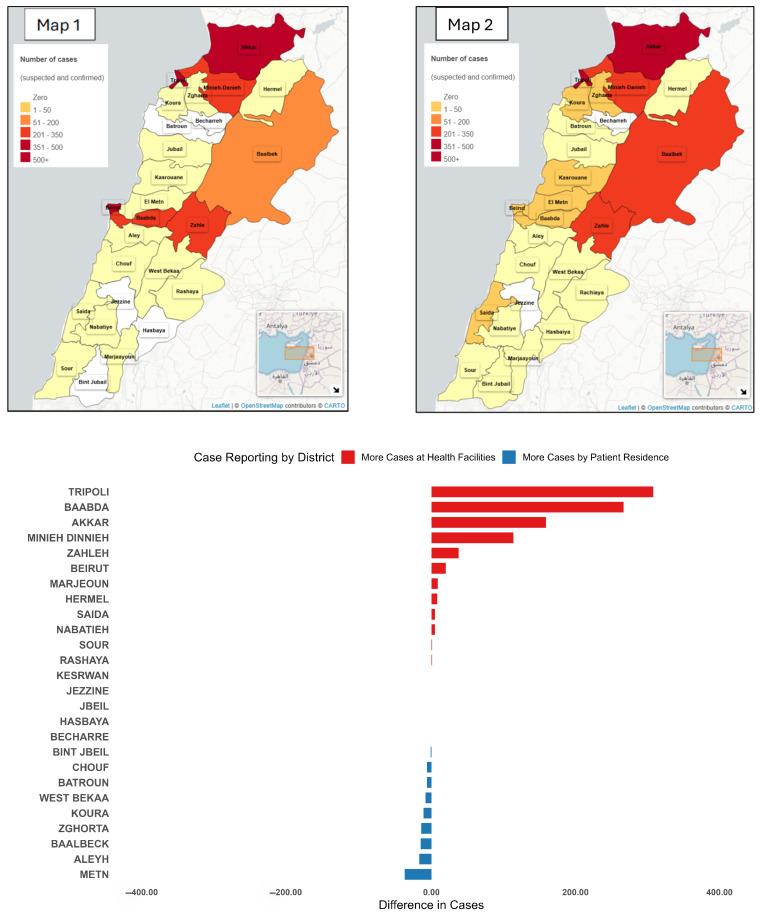
District-Level case differences: Health facility vs. patient residence, Lebanon, 2022–2023. Map 1 presents the incidence of cases notified by the location of reporting. Map 2 presents the incidence of cases by place of residence. The bar chart presents the difference between the number of cases notified by place of reporting and the number of cases notified by place of reporting.

Two separate maps were generated to represent the spatial distribution of cases: Map 1 based on the location of the reporting facility and Map 2 based on patients’ places of residence (Figure 4).

Among facility-entered cases, 5276 (88.6%) were reported from the same governorate as the patient’s residence, 467 (7.8%) were from an adjacent governorate, and 210 (3.5%) were from non-adjacent governorates. At the district level, 5028 cases (84.5%) were reported from the same district of residence, 512 (8.6%) from an adjacent district, and 413 (6.9%) from non-adjacent districts.

**Figure 5:**
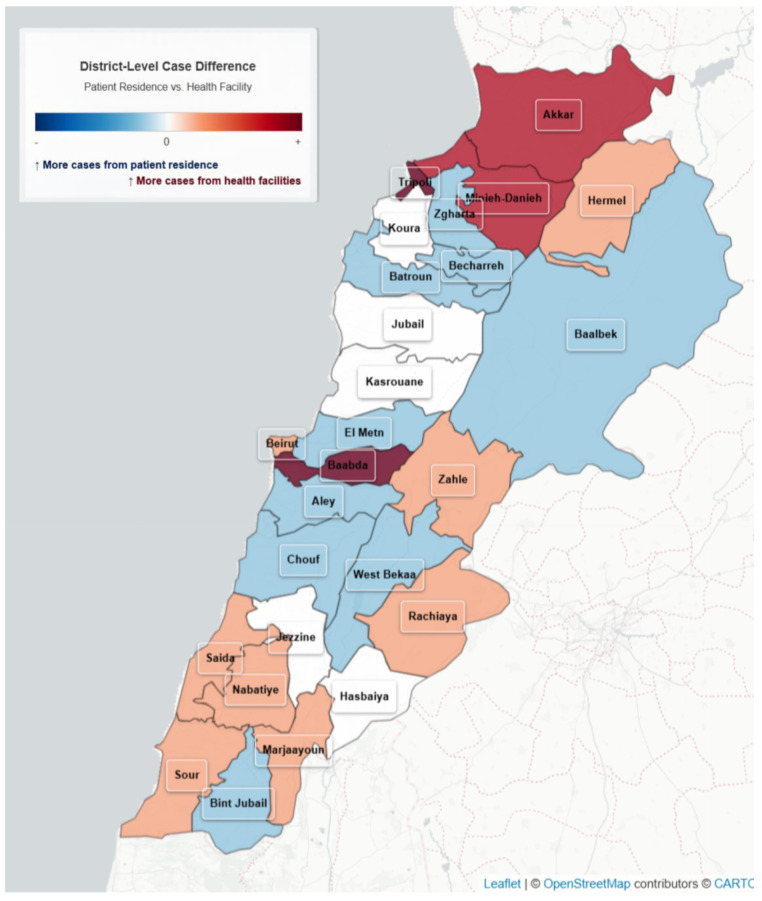
Variation in Cholera cases by reporting site and residence, Lebanon, 2022–2023.

We generated a choropleth map to display the magnitude and direction of differences between facility-based and residence-based case distributions across all districts (Figure 5).

### 3.4. Incidence Rate and Rapid Diagnostic Tests by District

**Figure 6:**
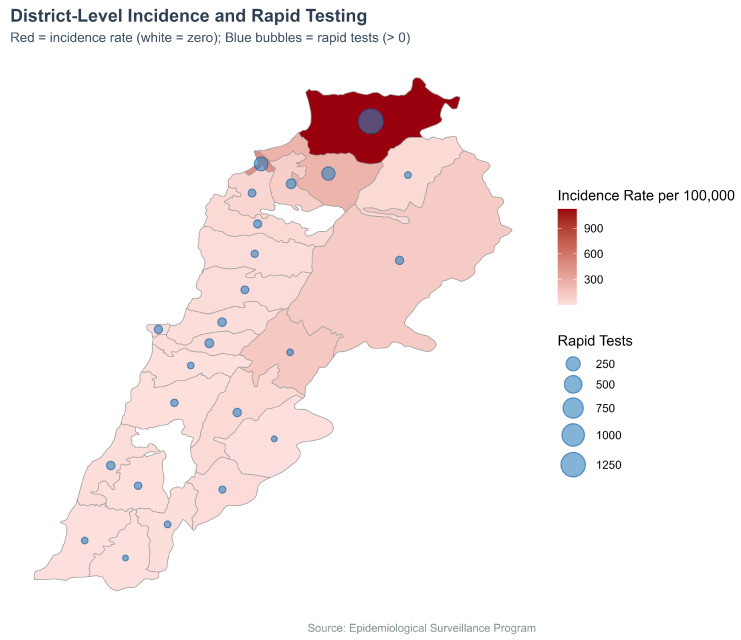
Cholera incidence (Choropleth) and RDTs (Bubble map) by district, Lebanon, 2022–2023.

We plotted the distribution of cholera incidence rates per 100,000 and the number of rapid diagnostic tests (RDTs) conducted across districts (Figure 6). Incidence was represented using a red gradient choropleth, while the number of rapid tests was shown as blue bubbles plotted at district centroids. Districts with zero reported cases appeared in white, and no bubbles were shown where no RDTs were conducted.

## 4. Discussion

The integration of R-based analytics into DHIS2 cholera surveillance during the 2022–2023 outbreak in Lebanon provided a deeper understanding of both data quality and surveillance performance. This process improved case classification, enhanced geographic mapping, and enabled better monitoring of RDT testing.

The first key observation concerns the application of the case definition of cholera. Initial reporting by health facilities classified 92.8% of cases as suspected and 7.2% as confirmed. However, retrospective reclassification using standardized clinical criteria and laboratory data showed 468 cases (5.8%) were confirmed by culture, 3239 (40.0%) met the clinical definition of suspected cholera, 3935 (48.6%) were unclassifiable due to insufficient clinical information, and 453 (5.6%) did not match the case definition. This finding underscores the need to align case definition of cholera across all levels, highlighting the need to reinforce case classification guidelines for health facility staff. Embedding simplified decision support tools within electronic systems could help front-line reporters classify cases more consistently in real time.

The second observation relates to laboratory testing. Two-thirds of cases had no documented laboratory results, and among those tested, only 5.8% were culture-confirmed, with an additional 2.2% testing positive by RDT. These figures reflect the WHO guidelines to test subsets of cases during the outbreak. Importantly, 2181 cases had negative laboratory results yet remained in the national line list. This reflected a decision to retain reported suspected cases during the outbreak, whatever laboratory result, avoiding confusion among the public and media when cases are removed after testing. This practice emphasizes the need for clear, standardized protocols for counting negative-tested cases that balance epidemiological classification with public communication needs.

The third observation addresses geographic data. The comparison of notified cases by reporting facilities with their residence provided important insights. High concordance was observed between place of reporting facility and residence at the governorate level, but notable differences remained at the district level, particularly in remote regions and Beirut suburbs. These differences likely reflect referral patterns and health-seeking behaviors and must be considered when interpreting disease spread or targeting interventions. During the outbreak, the mapping on place of residence was generated manually and shared on daily basis with the decision makers to guide the response activities.

Our experience aligns with recent international work indicating that while digital systems like DHIS2 can substantially improve timeliness and coverage of reporting, persistent challenges remain. For instance, in Ethiopia the integration of DHIS2 with climate-data early-warning systems showed potential but was hampered by infrastructure and data-quality limitations [12]. Similarly, in Ghana DHIS2-derived surveillance data were found to offer limited early-warning capability due to gaps in data completeness and consistency [13]. As our study demonstrates, adding an R-based data-cleaning step and retrospective re-classification can enhance the utility of surveillance outputs, albeit requiring additional technical capacity. These findings suggest that digital platforms offer significant advantages—real-time reporting, spatial mapping, system integration—but also require investment in human resources, data governance and analytical workflows to realise their full benefit [14].

## 5. Conclusions

The integration of R-based analytics into the DHIS2 cholera surveillance system in Lebanon significantly improved data completeness, case classification, and spatial interpretation. There is a need to explore embedding the R into the DHIS2. Such integration will allow automatic data cleaning for timely adequate case classification, re-assignment of geographical coding for automatic mapping on place of residence, and monitoring of laboratory testing across time and place.

To further strengthen surveillance, it is recommended to monitor the completeness of variables in reporting investigation forms, include clinical presentation in the investigation form (stage 2), ensure adequate training and awareness of health professionals on case definitions during outbreaks, and explore embedding R tools within DHIS2 to generate cleaned data for accurate and timely dashboards.

## Figures and Tables

**Table 1:** Proportion of missing values in selected variables before data cleaning in stage 1.

Variable	% Missing Values
Inpatients status	99.7%
Underlying Conditions	34.7%
Illness information	17%
Death outcome	34%

**Table 2:** Distribution of cholera cases by final classification outcome, Lebanon 2022-2023.

Case Classification	Number of Cases	Percentage (%)
Confirmed (culture)	468	5.8
Suspected (clinical)	3239	40
unspecified classification	3935	48.6
Unmatched	453	5.6

**Table 3:** Comparison of initial and final cholera case classification, Lebanon 2022–2023.

Classification Category	Initial DHIS2 (%)	After Reclassification (%)
Suspected	92.8	40.0
Confirmed (culture)	7.2	5.8
Unspecified classification	0	48.6
Unmatched	0	5.6

## Data Availability

The R script used for data cleaning and analysis in this study is publicly available on GitHub at (https://github.com/jounyabass/dhis2-r-cholera-analysis.git, Accessed on 1 January 2020). Mockup datasets are included to allow users to test and adapt the code. The actual surveillance data used in this study are owned by the Lebanese Ministry of Public Health and are not publicly available due to privacy and ethical restrictions.

## Abbreviations

The following abbreviations are used in this manuscript:

DHIS2	District Health Information System
ESU	Epidemiological Surveillance Unit
RDT	Rapid Diagnostic Test
MoPH	Ministry of Public Health
AWD	Acute Watery Diarreah
WHO	World Health Organization
